# Highlights from the 2016-2020 NEUBIAS training schools for Bioimage Analysts: a success story and key asset for analysts and life scientists

**DOI:** 10.12688/f1000research.25485.1

**Published:** 2021-04-30

**Authors:** Gabriel G. Martins, Fabrice P. Cordelières, Julien Colombelli, Rocco D’Antuono, Ofra Golani, Romain Guiet, Robert Haase, Anna H. Klemm, Marion Louveaux, Perrine Paul-Gilloteaux, Jean-Yves Tinevez, Kota Miura

**Affiliations:** 1 Instituto Gulbenkian de Ciência, Oeiras, Portugal; 2 Bordeaux Imaging Center (BIC), Université de Bordeaux - US4 INSERM, Bordeaux, France; 3 Institute for Research in Biomedicine (IRB Barcelona), Barcelona Institute of Science and Technology (BIST), Barcelona, Spain; 4 Crick Advanced Light Microscopy STP (CALM), The Francis Crick Institute, London, UK; 5 The department of Life Sciences Core Facilities, Weizmann Institute of Science, Rehovot, Israel; 6 BioImaging and Optics Platform (BIOP), Faculty of Life Sciences (SV), École Polytechnique Fédérale (EPFL), Lausanne, Switzerland; 7 DFG Cluster of Excellence “Physics of Life”, University of Technology TU, Dresden, Germany; 8 Science for Life Laboratory BioImage Informatics Facility and Department of Information Technology, Uppsala University, Uppsala, Sweden; 9 BioImage Analysis Unit, Institut Pasteur, Paris, France; 10 Image Analysis Hub, C2RT Institut Pasteur, Paris, France; 11 Université de Nantes, CNRS, INSERM, Nantes, France; 12 Université de Nantes, CHU Nantes, Inserm, CNRS, SFR Sante, Inserm UMS 016, CNRS UMS3556, Nantes, France; 13 Nikon Imaging Center, University of Heidelberg, Heidelberg, Germany; 14 Bioimage Analysis & Research, Heidelberg, Germany

**Keywords:** NEUBIAS, training schools, bioimage analysis, analyst, workshop, training materials, Analyst school

## Abstract

NEUBIAS, the European Network of Bioimage Analysts, was created in 2016 with the goal of improving the communication and the knowledge transfer among the various stakeholders involved in the acquisition, processing and analysis of biological image data, and to promote the establishment and recognition of the profession of Bioimage Analyst. One of the most successful initiatives of the NEUBIAS programme was its series of 15 training schools, which trained over 400 new Bioimage Analysts, coming from over 40 countries. Here we outline the rationale behind the innovative three-level program of the schools, the curriculum, the trainer recruitment and turnover strategy, the outcomes for the community and the career path of analysts, including some success stories. We discuss the future of the materials created during this programme and some of the new initiatives emanating from the community of NEUBIAS-trained analysts, such as the NEUBIAS Academy. Overall, we elaborate on how this training programme played a key role in collectively leveraging Bioimaging and Life Science research by bringing the latest innovations into structured, frequent and intensive training activities, and on why we believe this should become a model to further develop in Life Sciences.

## Introduction

**Figure 1.**
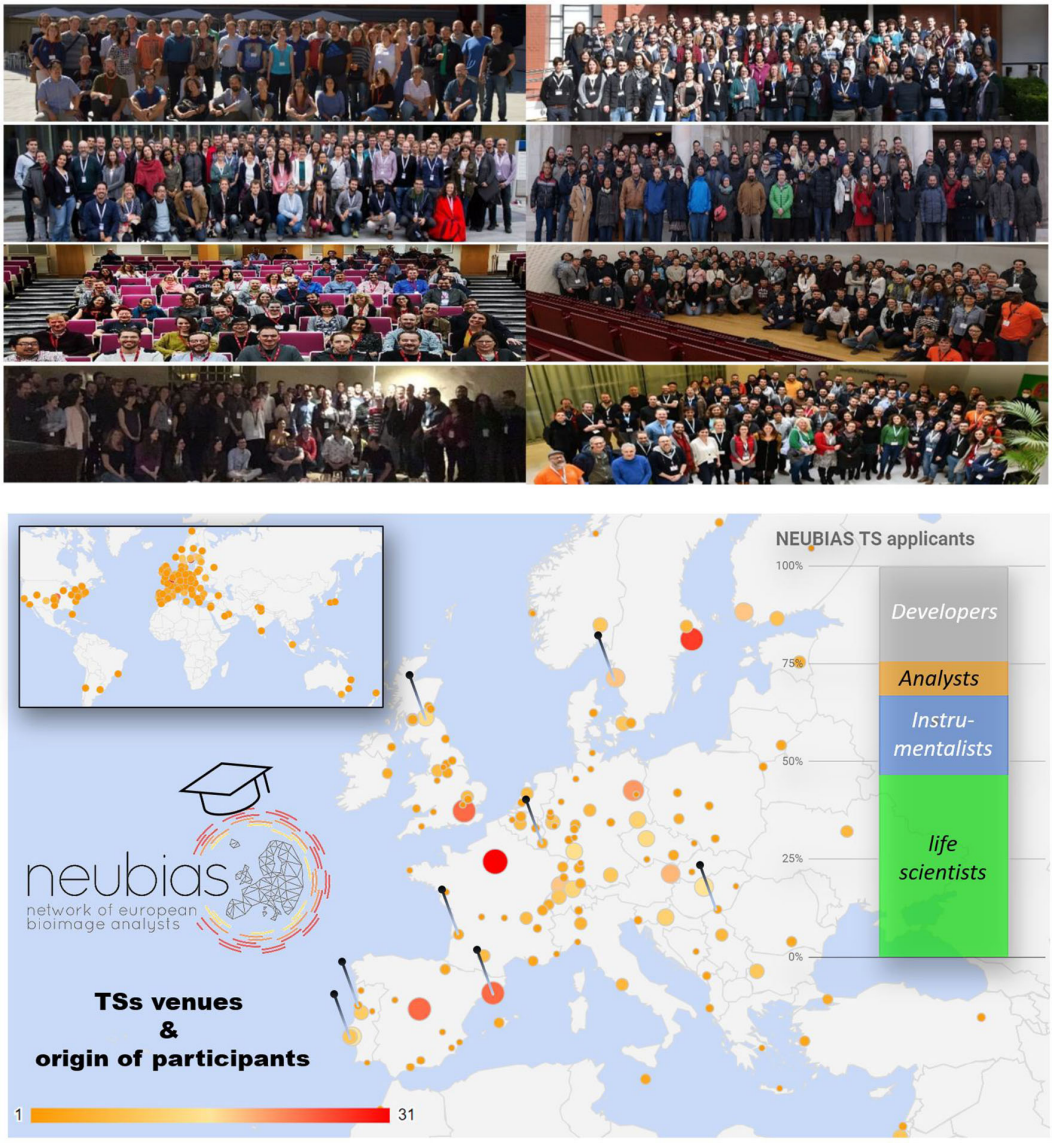
Overview of the 15 NEUBIAS training schools, which took place in eight venues between 2016 and 2020. Photos of participants: From left to right, top to bottom: TS1 for facility staff in Barcelona, Sept. 2016 (25 trainees); TS2 for early career researchers and TS3 for bioimage analysts in Oeiras Feb. 2017 (25+25 trainees + trainers); TS4 for early career researchers & TS5 for facility staff in Gothenburg, Sept. 2017 (25+25 trainees). TS6 for early career researchers and TS7 for analysts in Szeged, Jan. 2018 (23+35 trainees); TS8 for early career researchers & TS9 for facility staff in Edinburgh, Sept. 2018 (20+20 trainees); TS10 for early career researchers & TS11 for analysts in Luxembourg, Feb. 2018 (29+39 trainees); TS12 for early career researchers & TS13 for facility staff in Porto, Oct. 2019 (28+32 participants); and finally TS14 for early career researchers & TS15 for analysts in Bordeaux, Feb. 2020 (25+35 trainees). Map chart contains the locations of the different venues, the origin of the participants and a bar chart with the distribution of applicants based on their main expertise. See also Table 1 for more information about the schools, and online at http://eubias.org/NEUBIAS/training-schools/ for more information about the programme and venues. Authorization was obtained from the participants to capture and publicize group photos or photos taken during the event.

The NEUBIAS Training Schools (the “TSs” hereafter) have been developed as a series of 15 training events around Bioimage Analysis (BIA). These events took place between 2016-2020 over eight different venues throughout Europe ( Figure 1) and brought together four communities of experts: life-scientists, microscopists, software tool developers and bioimage analysts with the aim to train the researchers and the prospective trainers, and to promote the exchange of knowledge and experience between the four communities of experts. The TSs were highly successful and well praised by the over 400 trainees, and by the recruited community of over 100 invited organisers, trainers and speakers. The series led to the creation of numerous training contents on bioimage analysis from basic to advanced topics, which are archived in repositories and are being made accessible as “open access” via the “BioImage informatics index” tool ( http://biii.eu), also developed by the NEUBIAS community. The TS venues also led to the growth and strengthening of NEUBIAS as a professional network composed of bioimage analysts, software developers, instrumentalists and life scientists, with over 250 active members and over 5000 listed contacts. The interaction of the different professionals has enriched the bioimaging community hence fostering different disciplines to converge towards a common framework for BIA challenges and their research and technological needs. Here we review the conceptual design of the TSs content, how NEUBIAS tailored the level of the courses to the different needs of the aforementioned communities, and how the training programme has organically evolved over the years to cope with the tremendous pace at which BIA is developing. In the light of the TSs outcomes, the success stories and their immediate benefits for research, we discuss how instrumental international cooperation has been to support the Life Science community and the pressing need to develop a professional training programme in Bioimage Analysis in Europe, and beyond, so as to leverage local, regional, national or individual training initiatives that have substantially increased over the years of activity of the NEUBIAS network.

## Central concept(s) of NEUBIAS TSs

Bioimage analysis is an emerging research field, which has progressed alongside the fast-paced development of bioimaging techniques of the last couple of decades ( Meijering 2007; Schneider et al. 2012; Meijering et al. 2016; Miura & Tosi 2016; Peng et al. 2012). However, the incorporation of new BIA techniques has lagged behind, leading to a bottleneck in the progress of image-based research, for which there are several reasons. First, most life-scientists who work with bioimage data lack the formal training on BIA; although imaging and bioimage data recording are now common practice in the life-sciences, training with image data analysis is still practically nonexistent in most life-sciences courses. Secondly, there is often a communication barrier between BIA algorithm developers and most end-users (so far, these have been two separate communities), which limits the assimilation of novel techniques and leads to gaps in BIA. Compared to the conventional “image analysis” in Computer Science, which aims is to mimic the human visual perception and interpretation, i.e. Artificial Intelligence, BIA aims at the objective and quantitative measurement of biological phenomena avoiding human perception bias ( Miura & Tosi 2016). As visual recognition *per se* in most cases does not yield quantitative data, a new type of training that reaches beyond just the usage of software packages is required. Thirdly, modern scripting languages are necessary not only for automating, but also for properly and reproducibly documenting image data processing and quantitative measurements. For these reasons, more than just training about coding, the NEUBIAS TSs were designed to focus on training researchers about the “Art of Bioimage Analysis”, its scientific concepts and how to properly build and document effective workflows, using representative hands-on exercises.

The TSs were initially designed to address user requests following a 2015 survey among 2000 scientists in 32 countries worldwide ( Miura, 2021). The vast majority of early career researchers and imaging facility staff did not feel well prepared to extract quantitative data from bioimages; almost 60% reported BIA as the most difficult step in image-based research, with almost 80% considering it essential or very important. Of these, nearly half reported not having access to professional support in BIA, and most importantly, an estimated 60% of image data collected is not analysed, presumably because researchers lack the proper tools and skills for processing and analysis. Support and courses were identified as the most demanded resources for (68%) researchers who rely on BIA.

The TSs followed the pioneering steps of the successful “Bioimage Analysis Courses”, organized by NEUBIAS founders and seed of its creation, in Heidelberg (2013-2017) ^1^ as EMBL Master Courses, and in Barcelona (2013) ^2^ together with the 1st European Bioimage Analysis Symposium (EuBIAS), building on what was then already a community-driven effort. The uniqueness of these courses consisted in the training aimed at the quantification of biological processes, more than simply tutoring the usage of image analysis software packages and plugins. Through the financial support from H2020 COST Action CA15124 from 2016, NEUBIAS was able to expand the reach and attendees-throughput of these training efforts, and prepare a new programme for TSs targeting three main audiences and perceived levels of proficiency: the i) early career researchers, who need a solid and practical understanding of BIA concepts and techniques to carry on their biological research, and an overview of the existing tools and of the limits of ethics in BIA; the ii) imaging facilities staff, whose daily tasks involve supporting researchers in BIA and who require solid skills at designing and scripting effective and reproducible workflows; and the iii) bioimage analysts who need insights into advanced utilization and scripting of BIA resources, as well as the structured designing of novel workflows. More information about the concepts, content and training cohort of the NEUBIAS TSs can be found online ^3^^,^^4^.

The schools included sessions explaining the theoretical concepts behind the BIA techniques, a detailed explanation of a workflow exemplifying an application of the techniques, and fully documented practical exercises (with presentation slides, image datasets and exercise solutions/code). There were also guest-speaker plenary talks and ample moments for social interaction when participants (sometimes over 90) assembled at meals and coffee-breaks. Schools always concluded with “work-on-your-own-data” sessions with the support of trainers, analysts and developers, to provide the experience of a truly interdisciplinary environment. The “Analyst” schools had a somewhat different format, more workshop-style, and will be discussed in a subsequent section.

We believe the TSs were highly successful, having accepted 415 trainees out of over 900 applications from 43 different countries (see also Table 1). The rate of satisfaction averaged at 93%, and 10% of the trainees were accepted to progress through the different levels of the training programme. The seven “early career” schools trained 175 researchers (mainly life-scientists and instrumentalists), and despite being the most frequently organized they only reached 37% of the applicants, showing there is still a large community in need for training in BIA (see Figure 1 and Table 1 for applicant profiles and school statistics). The later-established online NEUBIAS Academy (explained below) was founded with this demand in mind. The four “facility staff” and four “bioimage analyst” schools, trained a total of 102 and 134 professionals, with acceptance rates of 46% and 62%, respectively. Most staff applicants considered themselves instrumentalists or analysts, and the analyst school applicants were either analysts or developers. By making this crucial effort to training professionals in imaging and analysis, instead of addressing primarily the massive demand from early career life-scientists, NEUBIAS sought to amplify the reach of the TSs, hoping that those professionals would be empowered by the training and more likely to have the time, the means and the motivation to train others locally - to guarantee this we often recruited trainees to train in subsequent schools. TSs organizers collected and catalogued numerous materials for reusing in future schools and workshops, contributing to the disseminating efforts. An excerpt of the topics and tools addressed in the schools is shown in Table 2. These include an impressive assortment of over 10 different open source toolboxes plus numerous plugins, tools and documented workflows.

**Table 1.** Profiles of the three target audiences that the NEUBIAS TSs tried to reach, their expertise levels, venues and brief statistics.

School for:	Profile	Expertise	Event	Statistics
**Early career researchers** **(basic)**	A researcher focused on a specific biological problem, and who needs to understand the basics of BIA and how to build workflows. Should not be a staff member with experience mostly with many different microscopy instruments. Should be early in the career (PhD student or with <6y after PhD). No *a-priori* knowledge required, but should have a clear need in order to advance in research, and an understanding of the needs of BIA. Has knowledge of some components, but little of the theory of BIA or is struggling to put them together as workflows.	beginner	TS2,4,6,8	37% acceptance rate (175/467); 25 trainees on average; 60% female trainees; 90% satisfaction; 13 trainers on average; 10-19 sessions/school.
**Early career researchers (intermediate)**	A researcher focused on a specific biological problem or a staff member who needs solid foundations of BIA and building workflows, but not yet ready for advanced scripting. Should be early in career (PhD student or with <6y after PhD). Should have already started using BIA tools with a purpose, and has images and a problem to bring to the school. Not focused on a specific component or tool (e.g. only needing to learn tracking or colocalization). One likely to be able to disseminate knowledge, and not yet a programmer/self declared macro expert.	intermediate	TS10,12,14	
**Facility staff**	A scientist or engineer working in the context of a bioimaging core facility and not focused on a specific project but providing regular support to multiple users and with potential to disseminate the training afterwards. One who is already performing BIA regularly and has a good understanding of the basics of processing & analysis. One who expresses a need to automate workflows, and not focused on a specific application (e.g. only in learning tracking or machine learning). One who is not already a programmer/self declared macro expert or too comfortable building workflows (an “analyst”).	professional	TS1,5,9,13	46% acceptance rate (102/222); 26 trainees on average; 44% female trainees; 92% satisfaction; 15 trainers on average; 12-19 sessions/school.
**Analysts**	A professional (or aspiring to be) that requires continuous training for utilizing novel algorithms, concepts, techniques and tools of BIA. One who has interest in networking with the community of other analysts: One who is able to code proficiently in at least one of the major programming languages used in BIA (Table II), and has great potential for becoming a dedicated analyst and in disseminating knowledge and organizing further training.	professional	TS3,7,11,15	62% acceptance rate (134/215); 34 trainees on average; 30% female trainees; 99% satisfaction; 9 trainers on average; 7-12 sessions/school.

**Table 2.** Examples of open-source toolboxes, components and workflows used during NEUBIAS TSs. These, and many others, are catalogued in Biii.eu with links to access the training materials from public repositories such as Zenodo and Github.

Toolboxes/languages	Used for training	Level(s)	NEUBIAS TSs
CellProfiler & Analyst	IA intro & workflows	basic, advanced	TS2,4,6,8,10,12,14
Drishti	3D visualization	basic	TS6
Icy	IA intro & workflows	basic, advanced, scripting	TS2,3,6
Ilastik	Workflows	basic, advanced	TS7,9,14
ImageJ/FIJI	IA intro, workflows & scripting	basic, advanced, scripting	all
KNIME	IA intro & workflows	basic	TS3,5
MatLab	IA intro, workflows & scripting	basic, advanced, scripting	TS1,3,5,6,9
OMERO	Workflows	basic	TS9
Python/scikit-image	Workflows & scripting	basic, scripting	TS5,13,15
QuPath	Workflows	basic	TS8,9
R	Workflows & analysis	basic	TS10,12
**Components**			
BigDataViewer	BIA of large datasets	basic	TS6,7,9,10,13
BigStitcher	IP of large datasets	advanced	TS9,10,12,14
ClIJ	IP workflows	basic, advanced	TS13,14
DeconvolutionLab	IP	basic	TS2
FigureJ	Figures for publication	basic	TS2,4,8,14
Filters & morpho. operators	IP techniques	basic, advanced, scripting	TS1,2,3,4,6,10,12,14
Imglib2/API	Development	advanced	TS11,13
SR-Tesseler	Analysis workflows	advanced	TS3
TrackMate/Mastodon	Tracking workflows	basic, advanced	TS5,6,7,8,10,13,14
**Workflows**			
3D Tubular Networks	Analysis workflows	advanced, scripting	TS1
Colocalization and clustering	Analysis workflows	basic, advanced	TS1,3,7,11,12,14
Machine Learning	IP workflows	basic, advanced, scripting	TS4,6,10,11,12,13,14,15
Particle analysis	Segmentation & analysis	basic, advanced	all
Segmentation	Analysis workflows	basic, advanced, scripting	TS1,2,4,5,6,79,10,12,13
SurfCut	IP workflows	advanced	TS15
Tracking	Analysis workflows	basic, advanced	TS1,3,5,6,7,8,9,10,12,13,14
**Other topics**			
Intro to bioimages	Data quality	all	TS2,12
Natural History of Fake Data	Ethics in BIA	all	TS2,4,6,7,10,12,14
P-value hacking	Ethics in BIA	all	TS15
Resources for Analysis	Review of tools	advanced	TS3,11,14
The Software Jungle	Review of tools	basic	TS14

## Choosing topics, trainers and trainees

The topics were selected by the organizers based on perceived needs of life-scientists, the trends on publications and results of TS evaluation surveys. The TS promoted the use of open-source tools and workflows, not just to promote FAIR principles but also to guarantee easier access and applicability for all participants. The programme privileged the exploring of different tools, including learning on the advantages of one toolbox *vs* others; for example alternatives to visualization and rendering of 3D datasets, which is still limited in the popular ImageJ/FIJI toolbox, but better in Icy ( de Chaumont et al. 2012) or Drishti ( Limaye 2012); or the handling of pyramidal-multi-megapixel image data easily done with QuPath (Bankhead 2017); or preparing pipelines for recursively analysing thousands of images using CellProfiler ( Carpenter et al. 2006), just to name a few. For those less familiar with scripting languages, TSs also showcased visual programming in KNIME ( Berthold et al. 2008) or Icy. The schools also included sessions where trainees learned how to integrate different toolboxes to tackle complex workflows which spanned the capabilities of one particular toolbox (for example, how to integrate ImageJ/FIJI with QuPath or Imaris or Icy).

During the unrolling of the programme there were shifts in the relative perceived importance of topics (seen also in the applications). For example, MATLAB became less requested and BIA with Python libraries and tools became a common request, which coincided with a significant increase in publication of machine/deep learning applications to BIA ( Meijering 2020). Handling of “big-data” appeared also on increasing demand, and both topics became prominent in TS after 2017-2018. The choice of topics was sometimes influenced also by the coincidence with the NEUBIAS Symposia and the availability of high-profile speakers. The schools also counted with the input from the NEUBIAS taggers, a community of BIA enthusiasts who gathered during the TS events to catalogue information about software and workflows and develop online tools to help bioimage analysts, which then fed into the TSs programme.

Applicants provided information that was used to match with an “ideal” profile outlined for each level of the TSs, as presented in Table 1. A committee of five evaluators read all applications anonymously and voted for acceptance based on the adequacy and preparedness of each candidate. This committee was typically composed of organizers and trainers of the current and previous events which helped maintain consistency in the selection process, and helped organizers tailor the priority topics and content of materials.

The NEUBIAS schools typically occurred in IT rooms, or in rooms prepared to accommodate for laptops (typically in the Analyst schools). Often, we paired trainees with different levels of experience to help, and all materials were prepared, tested and distributed upfront, to maintain a smoother training pace. A booklet was prepared for each school, and distributed in PDF format, with the names of participants and the synopsis of the sessions. The organizers communicated to the trainees what was the level of experience required, and sent reading materials, laptop preparation information and exercises in preparation for the schools.

Presentations often included two projections, one for the basic workflow description and a 2nd used by the main presenter to demonstrate “live” for demonstrations. A team of 5-10 “helper” trainers provided constant support, with a trainer-to-trainee ratio as high as 1:2 (see Table 1). Part of the helpers’ team was actually composed of recruited “trainers-in-training”. Some TSs experimented also with parallel sessions (taking advantage of the rich pool of trainers) which allowed trainees to choose topics/techniques more in line with their research needs.

After each school, all participants were invited to respond to a survey to provide feedback, on aspects such as duration, balance between theory and practice, preference for the tools and languages presented and methods used, other topics that should have been addressed and those that should be reduced. Each topic or session was also evaluated individually, so improvements could be made in subsequent editions. A report for each school was prepared and passed over to the next organizers of the event to try to improve and tailor the schools to the expectations of the previous trainees.

## The “Analyst” school - a novel concept of training in Bioimage Analysis

Bioimage analysis is an emerging field and there was clearly a lack of formal and dedicated training for “Bioimage Analysts”. However, as the field is evolving rapidly, those who are working already as experts in their institutes do require continuous training for utilizing novel algorithms, concepts, techniques, and tools. In addition, lateral communication among those experts needs to be promoted in the school-format to strengthen their own community. To design such a training school, the highly diverse educational backgrounds of Bioimage Analysts, ranging widely among almost all fields of the life sciences, hindered us from simply implementing an "advanced course" because each expert is differently advanced in various specific directions. For this reason, we made a simple assumption that the minimal common denominator of Bioimage Analysts is the ability to code proficiently in at least one of major programming languages used in bioimage analysis (for example, ImageJ macro, R, Java, Python, MATLAB, C, C++). This requirement also arises counterintuitively from the high quality of current BIA software, open-source or commercial. For many well established analysis workflows there are implementations in software that can be used readily by biologists. These tools are often accompanied by useful documentation and a good user-interface, and several new tools of this desirable quality emerge every year. We reasoned that the core added value of a bioimage analyst would be the ability to address analysis tasks for which an end-user accessible tool does not exist yet. Their work then involves combining image analysis components into new workflows, which requires at least some expertise in programming ( Miura et al. 2020).

This core task of analysts - creating new analysis pipelines - also shaped the following four concepts of the Bioimage Analysts School:

(1) **API Beating**, where we invited developers of interesting implementations of image processing/analysis algorithms (components) and asked for a fast-track navigation into the Application Programming Interface (API) and the practical hands-on to API’s entry points.
(2) **Workflow Deconstruction**, where we invited the authors of a paper with an interesting bioimage analysis workflow and asked them to explain how they solved their biological questions with their image analysis strategy. School participants examined the details of the solution, practiced code modification/migration trials with different components, refactoring, and also providing critical evaluations, which were all based on group coding ( Louveaux & Verger, 2021).
(3) **New algorithms**, where we invited the developer of the state-of-art bioimage analysis components and asked for an in-depth lecture.
(4) **Benchmarking**, where participants were asked to benchmark currently existing implementations.

In the last Analyst school (2020), an additional 5th concept was introduced: a module for utilizing the latest **statistical tool sets**, such as the Tidy Data Handling ( Bercowsky Rama, 2021) and the post-P value statistics ( Gómez-de-Mariscal, 2019). With the recent refinement of high-content imaging technologies (e.g. High Throughput Screening), statistical analysis of rich multidimensional image data has increasingly become an important aspect of analysis workflows, and still requires to be further developed and integrated so as to become a solid part of the Bioimage Analysts training concepts. As a by-product, the Analyst TSs were leveraged also to help aspiring analysts identify with the job and give them a sense of community and profession. Overall, the Analyst TSs pioneered a unique place for the training of BIA experts. Addressing these five concepts in a three and a half day event was often a challenge, and one that is likely to be addressed in the organization of future and potentially longer NEUBIAS Analyst schools.

Every year, all contents of the Analyst courses were freshly prepared based on the initial motivation to “train bioimage analysts with the latest knowledge and techniques”. For this reason, the Analyst schools often accepted some applicants more than once, which was not possible in the other schools, where trainees could only - and where in fact, encouraged to - progress to a more advanced level of the programme. This, and the fact that the Analyst schools were more “workshop style” and therefore requiring less trainers (in the Analyst schools trainees often were presenter themselves), allowed for a gradual increase in the number of accepted applicants per school (39 at the top). This was a focused effort to train analysts to boost the dissemination of NEUBIAS TS concept, and to establish a significant pool of professionals and an influential community of bioimage analysts.

## The training schools’ materials

The prioritized topics were identified for each school in preparatory meetings with the NEUBIAS working group that oversaw the TSs planning and invited the scientific organizers, who then took the previous programme and reports, suggested adaptations or improvements and invited more trainers. To promote continuity with the concepts and spirit of the TSs, some trainers, trainees and organizers from previous schools were invited. Efforts were made to create continuity between sessions, and to explore the possibility of featuring workflows that required a diversity of tools. Materials were stored in a cloud storage accessible by all those involved in the organization and training, and periodic meetings were done to discuss progress and consistency of the training materials being prepared.

With the collaboration of the trainers and speakers, NEUBIAS developed a collection of TS materials, which includes over 50 unique training sessions, fully documented with i) presentation slides ii) practical exercises with example data and iii) exercise solutions with instructions and code (“catch-up”). Currently, these materials are stored on an internal platform and are being catalogued and made accessible via the BioImage Informatics Index webtool ^5^, part of the recommended interoperability resources of ELIXIR ^6^ developed by NEUBIAS and whose content is maintained by the community as a crowd-sourced platform. These materials were openly shared among all participants during the TSs, who have reported to use them in their research and in local training efforts afterwards. Anyone interested in accessing and re-using these materials is encouraged to search in the Biii webtool, where NEUBIAS will keep the catalogue centralized and the most up-to-date links to the final repositories of these materials (see *Data availability*). There was an effort to try to create a consistent format and style for the presentation of the materials, including the slide formats, whenever possible. The organizers also experimented with video recording of some training sessions, when it was possible to fulfil GDPR compliance. Learning from this effort, “NEUBIAS Academy” was created in 2020 to offer recorded, yet compact, training modules for the community (more below).

Some of the TSs content was further refined and prepared in the form of book chapters included in the recently edited Bioimage Data Analysis Workflows books, with the first edition ^7^ freely available since 2019, and a second edition expected in 2021, while further publications based on the TSs training materials are envisioned to appear in the NEUBIAS F1000Research Gateway ^8^.

## Community building, follow-up and future perspectives

The NEUBIAS TSs, beyond the primary take-home training for selected participants, were highly regarded as a venue for getting to know “who is who” and what is “state-of-the-art” in BIA. They were also strategically organized, when possible, together with the NEUBIAS Symposia that most TSs trainees attended, and where they were further exposed to the latest innovations in techniques and networking opportunities. Overall, the level of trainer support during TSs was highly praised and quite unusual for this type of training, even though some students commented that the pace was at times fast or overwhelming, owing to the diversity of topics addressed ( Table 2). The organizers tried to balance the importance of exposing trainees to multiple tools and techniques, and of demonstrating how to explore the many existing software frameworks to tackle BIA problems and construct workflows. This is arguably another key aspect of NEUBIAS TSs, in contrast to other training initiatives with BIA modules but more oriented to specific biology-driven applications (e.g. 3D developmental imaging courses ^9^) or imaging-technology (e.g. Lightsheet EMBO courses ^10^). It was often explained that there is no “single tool for all tasks”, and that a well-prepared analyst should be ready to employ different tools.

The recruitment of trainees to serve as trainers in ensuing TSs is a significant outcome and has led also to several local training efforts that followed using the concept, methods and materials of the NEUBIAS TSs. Several trainees have reported becoming professional staff members or analysts themselves, and on the strong impact the attendance of the TS had on their career decision and CV (see, for example Box 1 containing “success stories”). Similarly to the recent establishment of national interest groups for Bioimage analysis by NEUBIAS stakeholders in their own country (e.g. IAFIG ^11^ in UK, Swissbias ^12^), new training initiatives derived from the NEUBIAS TSs community, such as a recently awarded EMBO course, focused on “advanced Methods in Bioimage Analysis ^13^. Robert Haase’s Youtube content ^14^, with over 1.000 subscribers, also benefited from NEUBIAS TS content. Other courses that followed on the footsteps of NEUBIAS TSs include the “Introduction for Image Analysis for life science” courses in Gothenburg ^15^, or the “advanced ImageJ Macro” course by the Max Planck Postdoc Net ^16^. Another initiative was recently funded under the auspice of EOSC Life (2nd Training Open call ^17^) for the organisation of “BioImage Analysis Defragmentation” Training Schools, which aim to continue training the new generation of bioimage analysts on workflow-based BIA with a focus on integrating methods for cloud-based and High-Performance computing applied to life sciences. This initiative also shows the current efforts of integration of the BIA community with other life science communities and infrastructures, and paves the way to better integration and reusability of bioimage analysis results within wider analysis workflows that include technologies other than imaging.

Box 1. **Selected NEUBIAS TSs success stories: Career path through the TSs program.**

**Anna Klemm.** While being a staff scientist at the Ludwig-Maximilians University (LMU) in Munich (Germany), she participated at TS1 and TS3 as a trainee. She then engaged as trainer in TS4, TS10, TS13, TS14 and as scientific organizer of TS8. She became a professional full-time bioimage analyst at the SciLifeLab BioImage Informatics Facility, Uppsala (Sweden) in 2018. Since January 2021 she has been Head of the facility.

**Marion Louveaux**. She attended TS7 as a trainee and became a trainer in TS10, 13 & 15 and co-organizer in TS10 & TS15. She is now an application specialist for the bioimage analysis software Icy at the Bioimage Analysis Unit, Institut Pasteur, Paris (France). “The Analyst school was a great opportunity to discover and connect to a network of professionals sharing the same interest”, she says, “and to consolidate an informal training in bioimage analysis. Later on, it also became a great opportunity to train others and co-organise training school events. This professional experience is still going on, with the NEUBIAS Academy, and the upcoming EOSC training school, and was probably key in securing a job with the Icy software team.”

**Nuno P. Martins**. Former imaging facility staff member at the Gulbenkian Institute of Science in Oeiras (Portugal), he attended TS1 & 7 as a trainee, and later became a trainer in TS2, and co-organizer of TS4. He is now pursuing a PhD in BIA at Max Planck Institute for Cell Biology and Genetics in Dresden MPI-CBG (Germany). He believes: “The TSs were a gateway to a community of interesting people with a lot of experience in BIA, advice and ideas on how to better do it”, and were instrumental for both the previous job at the facility and for the current PhD research.

**Robert Haase**. Formerly a core-facility staff scientist, and later postdoc at MPI-CBG in Dresden (Germany), he has become an analyst and tool developer ( Haase et al. 2020). He joined NEUBIAS TSs as a trainee in TS3, joined TS8 as trainer and acted as scientific organizer of TS13. Since 2021, he is a group leader of “Bioimage Analysis Technology Development” at the DFG Cluster of Excellence “Physics of Life” at the Technische Universität Dresden (Germany). His employment strategy follows the emerging career path promoted by NEUBIAS: In his group, life scientists with interest in BIA evolve towards becoming BIA experts, with career perspectives towards becoming BIA tool developers, lecturers or core-facility leaders.

But perhaps the most prominent follow-up of the NEUBIAS Training schools was the start of the “NEUBIAS Academy” ^18^. This new initiative stemmed from former trainers and trainees of the NEUBIAS TSs, with the aim of developing a community-driven framework for training in BIA. It has contributed to expand enormously the reach of the NEUBIAS training programme, especially during the COVID-19 pandemic, by starting to hold regular live webinars in 2020, and producing accessible online content. To date, NEUBIAS Academy has produced 28 webinars which attracted over 14,000 registrations from all continents. Webinars are recorded and publicly available on the NEUBIAS YouTube Channel ^19^ (>54,000 views, as of March 2021). While continuing to hold such webinars with increased focus onto thematic series (e.g. “Big Data Analysis Series”, “Correlative and Multimodal Analysis” ^20^), the NEUBIAS community envisions to resume in-person training as satellite events of the NEUBIAS conference, which has become a reference meeting point for the bioimage analysis community. The recent enthusiasm and community engagement witnessed with the NEUBIAS Academy webinars show that the interest and demand for training in BIA, as well as the community, are still growing. We believe this interest has been expanded by the success stories of the TSs, which were carried worldwide by the hundreds of participants. The fact that the TS reached such a widespread community (see Figure 1) certainly helped raise awareness among life science researchers that this training is fundamental, but more importantly, that it is available and supported by a large and active community, ready to engage and to supply solutions.

While local and national initiatives are slowly coming into place in several countries in Europe or overseas ^21^, the consolidation of Bioimage Analysis as a key research field able to leverage bioimaging resources and life science research will require in the coming years a sustained international coordination and new funding mechanisms to continue building similar training capabilities, at high levels of quality *and* quantity to match the current needs. A similar effort developed recently is the I2K event “From Images to Knowledge with ImageJ & Friends” (2018; 2020 ^22^), with a similar community-building ethos, and a large audience of BIA enthusiasts and developers. Given the increase in demand for BIA training, and the trend for remote training in times of social distancing and climate change, we envision future TSs in mixed format to explore the availability of online resources and webinars, with concurrent “in-person” trainings organized locally at different locations worldwide. The coordinated organization of these events would include shared topics, speakers/presenters, training materials, concepts, training practice and discussions following the NEUBIAS TS style. This promotes international cooperation, standardized methods and recognized levels of expertise, broader dissemination of training efforts, empowers local initiatives to contribute and benefit from the resources of a more global community, and will build on the spirit of a community-gathering that has become a hallmark, and highly praised, characteristic of NEUBIAS events. Ultimately, we have raised the interest and the level of awareness that hopefully will contribute to the inclusion of formal training in BIA in life-sciences curricula, at least at a basic level. The NEUBIAS community is hereby open to participate in efforts to create new higher-education curricula and teaching contents on BIA.

## Data availability

NEUBIAS TS materials are catalogued and made accessible via the BioImage Informatics Index webtool ( http://biii.eu), where NEUBIAS will keep the catalog centralized and the most up-to-date links to the final repositories of the materials (Zenodo and GitHub, for those that are complete and ready to be shared publicly via a CC-BY 4.0 Creative Commons License).

## Footnotes

^1^

http://wiki.cmci.info/shared/bias2013 and other links thereafter.

^2^

http://eubias2013.irbbarcelona.org/bias-2-course.

^3^

http://neubias.org/NEUBIAS/venue/neubias/training-wg2/.

^4^

http://neubias.org/NEUBIAS/training-schools/.

^5^

http://www.biii.eu.

^6^

https://elixir-europe.org/platforms/interoperability/rirs.

^7^

https://link.springer.com/book/10.1007/978-3-030-22386-1.

^8^

https://f1000research.com/NEUBIAS.

^9^

https://meetings.embo.org/event/18-developmental-imaging.

^10^

https://meetings.embo.org/event/18-lsm.

^11^
Image Analysis Focused Interest Group of the Royal Microscopy Society: https://iafig-rms.org/about.

^12^
SwissBIAS: The Swiss BioImage Analysts’s Society. https://swissbias.ch/.

^13^

https://www.embl.org/about/info/course-and-conference-office/events/bia21-01/.

^14^

https://www.youtube.com/playlist?list=PL5ESQNfM5lc7SAMstEu082ivW4BDMvd0U.

^15^

https://www.gu.se/en/core-facilities/graduate-course-introduction-in-image-analysis-for-life-science.

^16^

https://www.bioimagingnet.mpg.de/events/26607/6105.

^17^

https://www.eosc-life.eu/services/training/.

^18^

https://neubiasacademy.org/.

^19^

https://www.youtube.com/neubias.

^20^
In partnership with COMULIS, COST Action CA17121, https://www.comulis.eu/.

^21^
The Center for Open Bioimage Analysis: https://openbioimageanalysis.org/.

^22^

https://www.janelia.org/you-janelia/conferences/from-images-to-knowledge-with-imagej-friends.
