# Supplementary material for: Highlights from the 2016-2020 NEUBIAS training schools for Bioimage Analysts: a success story and key asset for analysts and life scientists
Source: F1000Res. 2021 Apr 30;10:334. [Version 1] doi: 10.12688/f1000research.25485.1 (PMC8215561; doi:10.12688/f1000research.25485.1)
Supplement: Peer review history

# Peer review: Reviewer response for version 1

**DOI:** 10.5256/f1000research.28124.r84283

**Published:** 2021-06-18

**Authors:** Kevin Eliceiri

**Affiliations:** 1 Laboratory for Optical and Computational Instrumentation, Center for Quantitative Cell Imaging, University of Wisconsin at Madison, Madison, WI, USA

The authors present an important aspect of the NEUBIAS, the European Network of Bioimage Analysts, the training schools. These schools were a central educational tool of NEUBIAS and were done as 15 events around biomaging analysis. During the four year term of the program, over 400 trainees attended, and involved over 100 invited organizers, trainers and speakers. The paper does a nice job of outlining the rationale behind the innovative three-level program of the schools, the curriculum, the trainer recruitment and turnover strategy, the outcomes for the community and the career path of analysts, including some success stories. There are important lessons to be shared from this program that could guide future such events. As such the enthusiasm for this article is very high.

 The only missing element in this reviewer's opinion is some sort of discussion in surveying of how the attendees were directly impacted. It is stated the attendees well received this program and some success stories are given but no direct metrics. For example, it was stated that some survey of student knowledge going into the program (pre-training) was assessed but there was no mention of student knowledge gained from the school (post training). This is a lost opportunity. If available it would be good to have a discussion and/or table to see in what areas attendee's knowledge of image analysis improved.  If this data is not available some discussion of future need for concept evaluation would be helpful

Is the topic of the opinion article discussed accurately in the context of the current literature?

Yes

Are arguments sufficiently supported by evidence from the published literature?

Yes

Are all factual statements correct and adequately supported by citations?

Yes

Are the conclusions drawn balanced and justified on the basis of the presented arguments?

Yes

Reviewer Expertise:

image informatics, image analysis, imaging, cell biology

I confirm that I have read this submission and believe that I have an appropriate level of expertise to confirm that it is of an acceptable scientific standard.

# Peer review: Reviewer response for version 1

**DOI:** 10.5256/f1000research.28124.r84282

**Published:** 2021-05-07

**Authors:** Thorsten Wohland

**Affiliations:** 1 Department of Biological Sciences, National University of Singapore, Singapore, Singapore; 2 Department of Chemistry, National University of Singapore, Singapore, Singapore; 3 Center for Bioimaging Sciences (CBIS), National University of Singapore, Singapore, Singapore

Martins *et al.* report on the outcome of NEUBIAS, a COST Action to create a network of European bioimage analysts. NEUBIAS has over the years run training schools at different levels from beginners to professionals and helped create a community of bioimage analysts well beyond the borders of Europe that connects between researchers in and practitioners of bioimage analysis. NEUBIAS has created very effective training schools that not only train interested participants but “trains the trainers”, which led to several local efforts to provide training courses in different countries using the same concepts that NEUBIAS used. In addition to training and networking, NEUBIAS has been involved in the creation and publication of bioimage analysis protocols and workflows and has helped train bioimage tool developers and facility managers. This report explains the core concepts of NEUBIAS and how it achieved its goals. This can function as a blue print for other communities with similar needs for networking and the dissemination of specialist knowledge in a diverse community to create well sought after experts in new upcoming fields.

Is the topic of the opinion article discussed accurately in the context of the current literature?

Yes

Are arguments sufficiently supported by evidence from the published literature?

Yes

Are all factual statements correct and adequately supported by citations?

Yes

Are the conclusions drawn balanced and justified on the basis of the presented arguments?

Yes

Reviewer Expertise:

Biophysical Fluorescence

I confirm that I have read this submission and believe that I have an appropriate level of expertise to confirm that it is of an acceptable scientific standard.
